# HDL Cholesterol Is Remarkably Cardioprotective Against Coronary Artery Disease in Native Hawaiians and Pacific Islanders

**DOI:** 10.1016/j.jacadv.2025.101741

**Published:** 2025-05-02

**Authors:** Austin Szatrowski, Zane Maggio, Bohdan Khomtchouk

**Affiliations:** a The College of the University of Chicago, Chicago, Illinois, USA; b Department of Biomedical Engineering and Informatics, Luddy School of Informatics, Indiana University, Indianapolis, Indiana, USA

**Keywords:** cardioinformatics, coronary artery disease, high-density lipoprotein, Native Hawaiians and Pacific Islanders, risk prediction

## Abstract

**Background:**

High-density lipoprotein cholesterol (HDL-C) is inversely associated with cardiometabolic risk and exhibits nonlinear effects at extreme levels. Cardiometabolic diseases are a leading cause of death and are particularly prevalent among Native Hawaiian and Pacific Islanders (NHPIs).

**Objectives:**

This study characterizes HDL-C’s association with coronary artery disease (CAD), major adverse cardiovascular events (MACE), and type 2 diabetes (T2D) in NHPIs compared to the general population.

**Methods:**

Using electronic health record data from the National Institutes of Health All of Us Research Program, we applied Cox proportional hazards models to compare HDL-C’s protective effects on CAD, MACE, and T2D between 261 NHPIs and the remaining cohort (n = 188,802). Models were adjusted for key confounders, and restricted cubic splines were used to assess nonlinear risk dynamics.

**Results:**

Tracking individuals across 10,534,661 person-years (mean age 55.7 ± 15.8 years, 38% male), HDL-C was more strongly associated with reduced CAD risk in NHPIs (HR: 0.32; 95% CI: 0.19-0.54) than in the general cohort (HR: 0.57; 95% CI: 0.56-0.58). A marginally stronger association was observed for MACE (NHPI HR: 0.40; 95% CI: 0.23-0.71 vs general HR: = 0.54; 95% CI: 0.53-0.56), while T2D associations were similar. Spline analysis indicated that low HDL-C increases risk for both CAD and T2D in NHPIs.

**Conclusions:**

HDL-C’s protective role against cardiometabolic diseases is more pronounced in NHPIs, particularly for CAD. These findings support further investigation into tailored clinical assessments for this population.

High-density lipoprotein (HDL) plays a critical role in cardiometabolic health by mediating reverse cholesterol transport, aiding reversal of atherosclerosis, and reducing inflammation.^1^, ^2^, ^3^, ^4^ Despite its potential, efforts to harness HDL as a therapeutic target for cardiovascular disease (CVD)—the world’s leading cause of death—have been largely unsuccessful to date.^5^ Indeed, both clinical trials and Mendelian randomization analyses have shown that raising HDL (as measured by HDL cholesterol [HDL-C] levels) does not necessarily correlate with reduced CVD risk.^6^ However, it remains a powerful tool for risk prediction, including as a component of the American Heart Association/American College of Cardiology Pooled Cohort Equations and is increasingly implicated in non-CVDs including type 2 diabetes (T2D).^6^^,^^7^

At particularly high risk for cardiovascular and metabolic disease are Native Hawaiian and Pacific Islander (NHPI) populations. In this group, prevalence of hypertension, hyperlipidemia, obesity, coronary artery disease (CAD), T2D, and stroke is 30% to 100% higher than in the general population, often with much earlier onset.^8^, ^9^, ^10^, ^11^, ^12^, ^13^, ^14^, ^15^ Compounding these substantial health differences is the minimal representation of NHPI in biobanks and large-scale epidemiological database studies, owing to their relatively small population size and challenges in health care access.^16^ Without representation, they are often left unconsidered when clinical practice guidelines and risk prediction tools are developed.^17^

Given these considerations, we conducted a study of HDL-C’s protective effects among NHPIs in the new and expansive National Institutes of Health *All of Us* Research Program database cohort using its rich archive of electronic health record (EHR) data sets to longitudinally track lipid levels and disease diagnoses across participant lifetimes (Central Illustration). Given that studies establishing HDL-C as a biomarker for CVD did not include NHPI participants, we hypothesized that its effect might be different among these population groups. First, we sought to quantify the magnitude of the association between high HDL-C and CAD, major adverse cardiovascular events (MACE) (which is a composite of myocardial infarction, sudden cardiac death, and ischemic stroke), and T2D using Cox proportional hazards regressions, among both NHPI and non-NHPI individuals. Second, to characterize these associations in more detail, we constructed restricted cubic spline curves for HDL-C’s effect on CAD and T2D risk, again for NHPI and non-NHPI individuals.

**Central Illustration:**
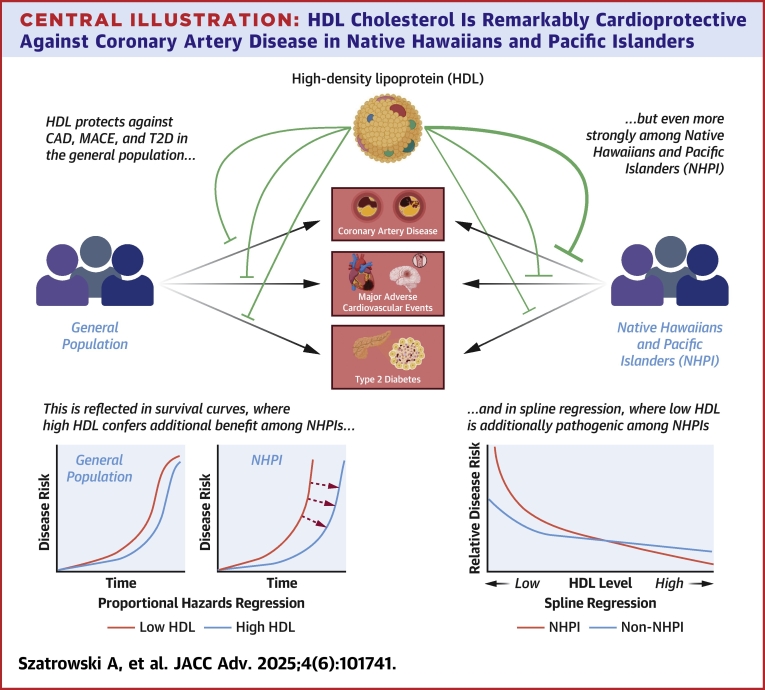
HDL Cholesterol Is Remarkably Cardioprotective Against Coronary Artery Disease in Native Hawaiians and Pacific Islanders Abbreviations as in Figures 1 and 3.

## Methods

### Cohort construction and covariates

All measurements and diagnoses were extracted from the EHR data coded and made available through the NIH *All of Us* Research Program’s Researcher Workbench platform (https://workbench.researchallofus.org, Curated Data Release v8, collected through October 1, 2023). Participants without shared EHR data were excluded. All data analysis was conducted in accordance with *All of Us* privacy and research ethics policies, and separate internal Institutional Review Board approval was not deemed necessary for conducting this study.

**Figure 1:**
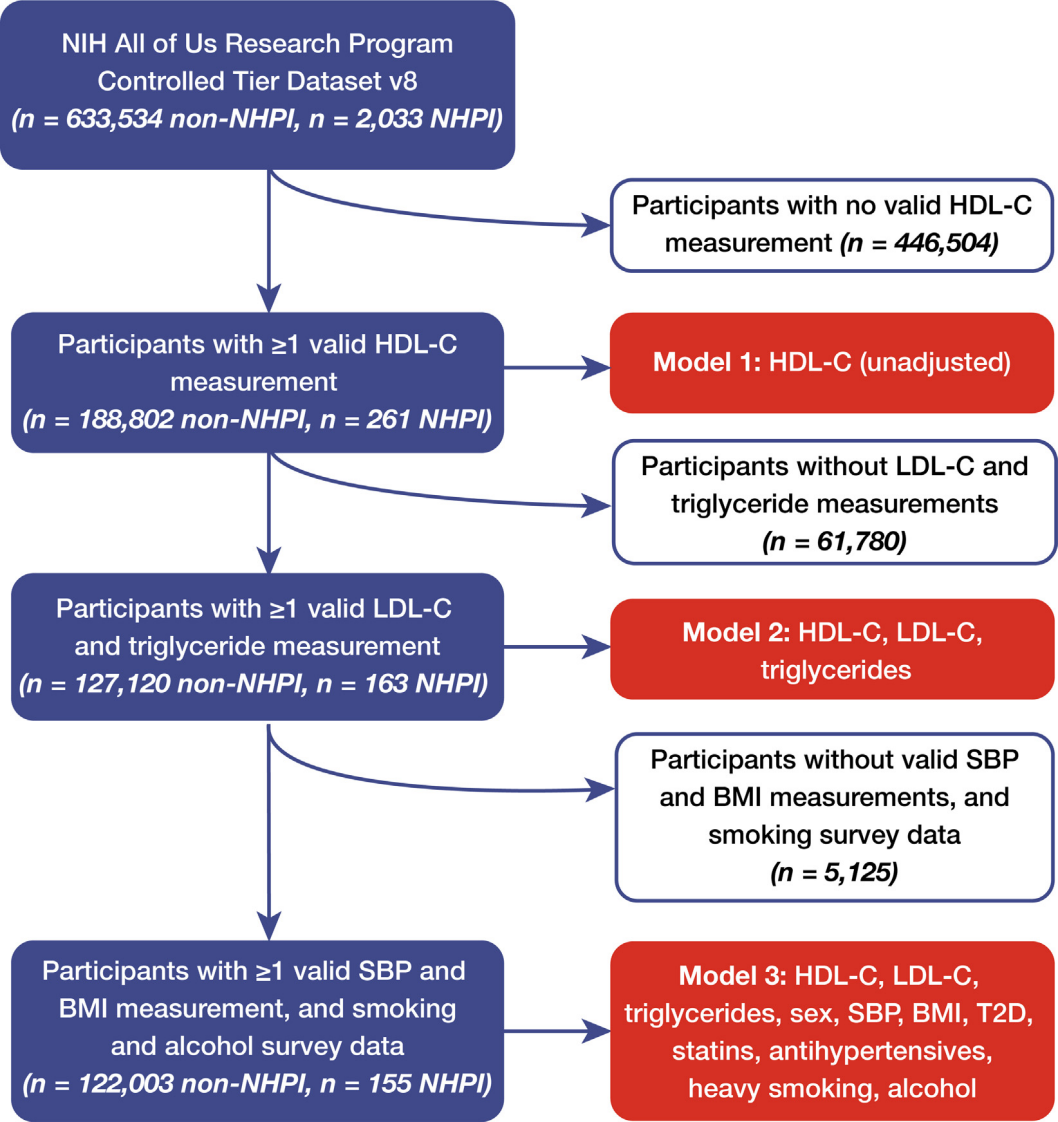
Flowchart for the Creation of the Study Cohort BMI = body mass index; HDL-C = high-density lipoprotein cholesterol; LDL-C = low-density lipoprotein cholesterol; NHPI = Native Hawaiians and Pacific Islander; SBP = systolic blood pressure; T2D = type 2 diabetes.

Our initial cohort consisted of all individuals in *All of Us* with at least one valid HDL-C measurement (*n* = 189,063), which we dichotomized as high or low, relative to the median level of 51 mg/dL. We then added diagnostic history for CAD, MACE, and T2D, and adjudicated outcomes using International Classification of Diseases 9, International Classification of Diseases 10, and Systematized Nomenclature of Medicine codes (Supplemental Table 1).^18^ We did not attempt to adjudicate diagnoses using proxies like coronary revascularization procedures or blood glucose. This constituted Model 1 (Figure 1).

For Model 2, we added first-available measurements of low-density lipoprotein cholesterol (LDL-C) and triglycerides as covariates; for Model 3, we added to Model 2 sex, body mass index (BMI), systolic blood pressure (SBP), diagnosis of T2D, prescription records for statins (ATC C10AA) and antihypertensive medications (ATC C02), and self-reported history of heavy drinking (Figure 1). With the addition of covariates, some individuals were removed due to missing data; rates of exclusion were comparable across the NHPI and non-NHPI cohorts (Figure 1). Additionally, those without recorded statin or antihypertensive prescriptions were assumed not to have taken them (Figure 1). We preselected these covariates before the analysis was performed, using a well-established list from previous research on HDL’s protective effects.^15^

We generated a variable for NHPI status (NHPI or non-NHPI) using participant self-identification from the *All of Us* survey questionnaire (Supplemental Figure 1), and in accordance with the National Academies and American Heart Association recommendations, participants are referred to by their NHPI status throughout the manuscript in the same manner.^19^^,^^20^

### Quality control

Given that EHR data often contain measurements reported in different units or data points recorded in different ways, we performed quality control on each measurement to ensure complete data harmonization. Measurements were standardized to mg/dL for all lipids, kg/m^2^ for BMI, and mm Hg for blood pressure. Extreme values, far outside physiological ranges due to apparent measurement or reporting errors or miscoding of units, were excluded from all quantitative measurements.

### Statistical analysis

We retrospectively followed a sample of 188,802 individuals using EHR data for a cumulative 10,534,661 person-years and reported baseline characteristics as mean ± SD or n (%), with Student’s *t*-test or chi-square for comparison tests (Table 1). Using the self-identified race of the participants from the *All of Us* survey questionnaire, we extracted an NHPI sample for comparison to the non-NHPI individuals in the cohort. We used univariate (Model 1) and multivariate Cox regression models (Model 2 and Model 3) to identify differences in HDL-C’s protective effects (dichotomized at 51 mg/dL, using first available measurement) against CAD, MACE, and T2D. Time 0 was defined as birth, and individuals were followed until the time of the first event or time of *All of Us* survey completion, whichever occurred first. Results are reported as the HR with 95% CI. Proportional hazards model assumptions for all outcomes were assessed using Schoenfeld residuals as implemented in the cox.zph() function from the R package *survival*.^21^

**Table 1:** Physical, Vital, and Clinical Characteristics of Study Cohort

	All Non-NHPI (n = 188,802)	NHPI (n = 261)	*P* Value
Male	52,793 (38.3%)	74 (43.0%)	0.29
Age (y)	60 ± 16	57 ± 16	0.0000344
HDL-C (mg/dL)	54 ± 17	51 ± 17	0.000334
LDL-C (mg/dL)	103 ± 35	199 ± 39	0.656
Triglycerides (mg/dL)	123 ± 72	130 ± 88	0.000885
Systolic blood pressure (mm Hg)	127 ± 18	128 ± 18	0.113
Body mass index (kg/m^2^)	39.99 ± 7.46	32.05 ± 8.41	0.0000064
Heavy smoking history	29,140 (24.1%)	24 (16.3%)	0.145
Statins	56,892 (47.1%)	65 (45.6%)	0.281
Antihypertensives	30,037 (24.9%)	41 (27.9%)	0.00582

Value are n (%) or mean ± SD. Quantitative measures are reported to the precision available in the original data set. *P* values are reported from chi-square tests for binary variables and *t*-tests for continuous variables, comparing all non-NHPI to NHPI. Each value is extracted from the data set used for the model in which the value was included (eg, statin usage for Model 3), so percentages may not exactly align with overall counts.

HDL-C = high-density lipoprotein cholesterol; LDL-C = low-density lipoprotein cholesterol; NHPI = Native Hawaiians and Pacific Islander.

Next, we fit splines to understand nonlinear risk dynamics. A spline is a piecewise function consisting of local polynomials whose coefficients, in the case of regression analysis, are optimized for goodness-of-fit to the data. This makes them an especially useful tool for understanding nonlinear and nonmonotonic trends in data, such as the one that HDL-C is widely understood to have with CVD risk and mortality.^6^

To ensure the robustness of our spline estimates, we analyzed HDL-C values exclusively within the 20 to 80 mg/dL range—the core interval where the bulk of our data reside. Within this range, we computed a kernel-smoothed coefficient of variation (CV) using a Gaussian kernel with a 3 mg/dL bandwidth. The resulting continuous CV curve allowed us to assess local measurement variability and identify HDL-C values with acceptable stability by applying a CV threshold of 0.1. By focusing solely on the 20 to 80 mg/dL interval, we minimized heteroscedasticity and enhanced the reliability of our restricted cubic spline estimates in capturing the nonlinear risk dynamics associated with HDL-C. The kernel-smoothed CV estimates for both NHPI and all non-NHPI are reported in Supplemental Figure 5.

### Data and code availability

Due to the stringent privacy policies of the NIH *All of Us* Research Program, which prohibit the dissemination of individual-level data, the authors are unable to share the underlying data utilized in this study. However, *All of Us* Research Program data are available to researchers upon request (researchallofus.org). All data management and analysis was conducted in the Google Cloud Platform servers of *All of Us* using the R programming language^22^; standard *tidyverse*^23^ tools were used for data management, survival analysis was conducted using *survival*^21^^,^^24^ and visualized using *survminer*.^25^ Splines were fitted using *rms*^26^ and plotted with *ggplot2*.^27^

## Results

We found that, across our 3 covariate models, HDL-C was remarkably protective against CAD among NHPIs compared to the rest of the population, while this effect was weaker for the composite measure MACE and null for T2D. Our spline regression analysis then revealed that unlike the general population, where the risk steadily decreases with increasing HDL-C, the relationship between HDL-C levels and CAD and T2D risk among NHPIs is notably complex and nonmonotonic.

### Cohort characteristics

Our cohort for analysis comprised 261 NHPI individuals and 188,802 non-NHPI individuals, of which 40.9% and 39.0%, respectively, were male. The mean age in both cohorts was 55.7 ± 15.8 years. Levels of HDL-C, LDL-C, and triglycerides in both cohorts were all comparable, as were SBP and heavy smoking rates (Table 1).

### Proportional hazards regressions

**Figure 2:**
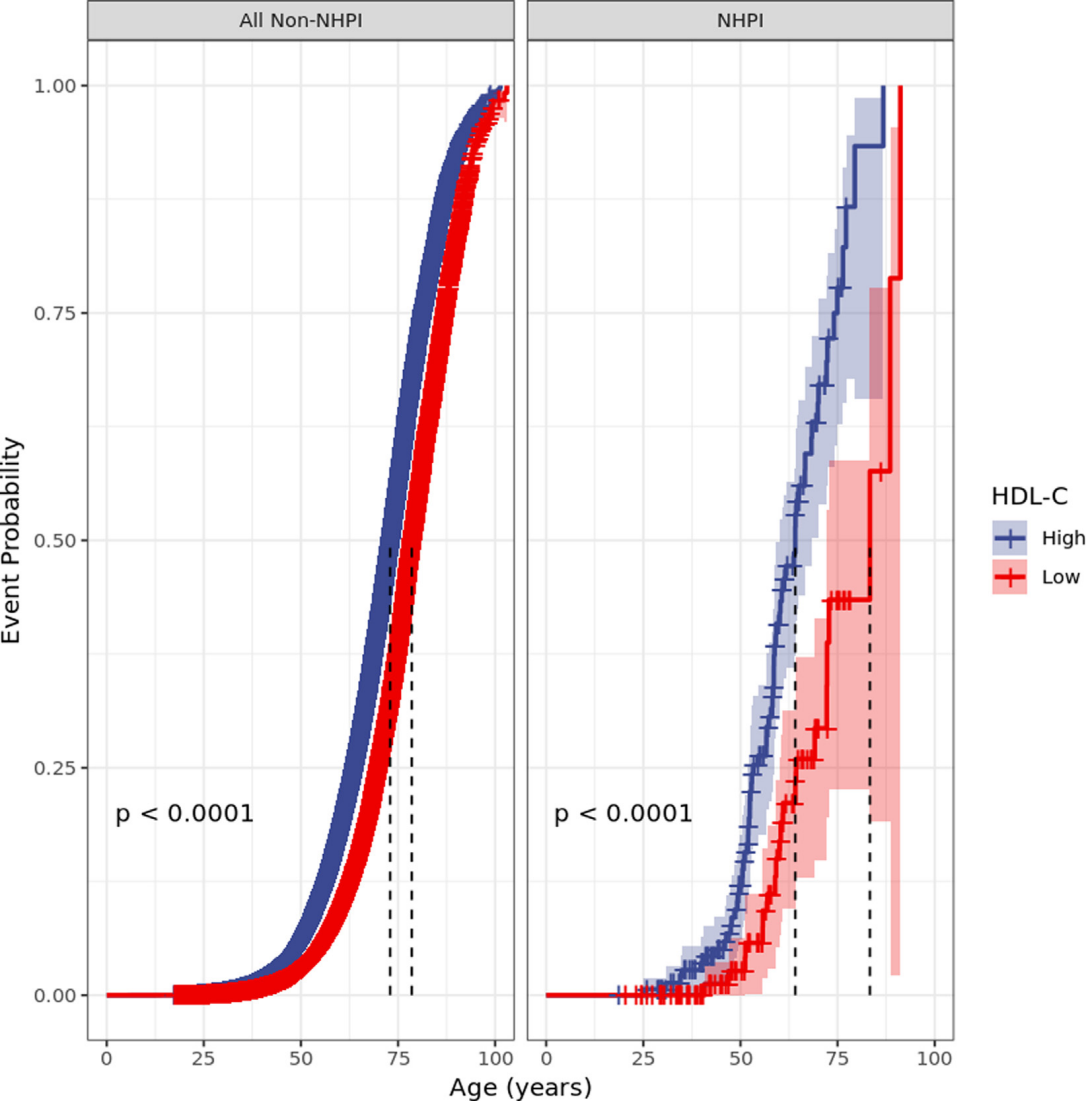
Causal-Specific Proportional Hazards Curves for High-Density Lipoprotein Cholesterol in the Non-Native Hawaiian and Pacific Islander and Native Hawaiian and Pacific Islander Cohorts Outer ribbons, 95% CIs; dashed lines, per-group median survival. *P* values from likelihood ratio test for comparing HDL-C within each group. Abbreviation as in Figure 1.

**Figure 3:**
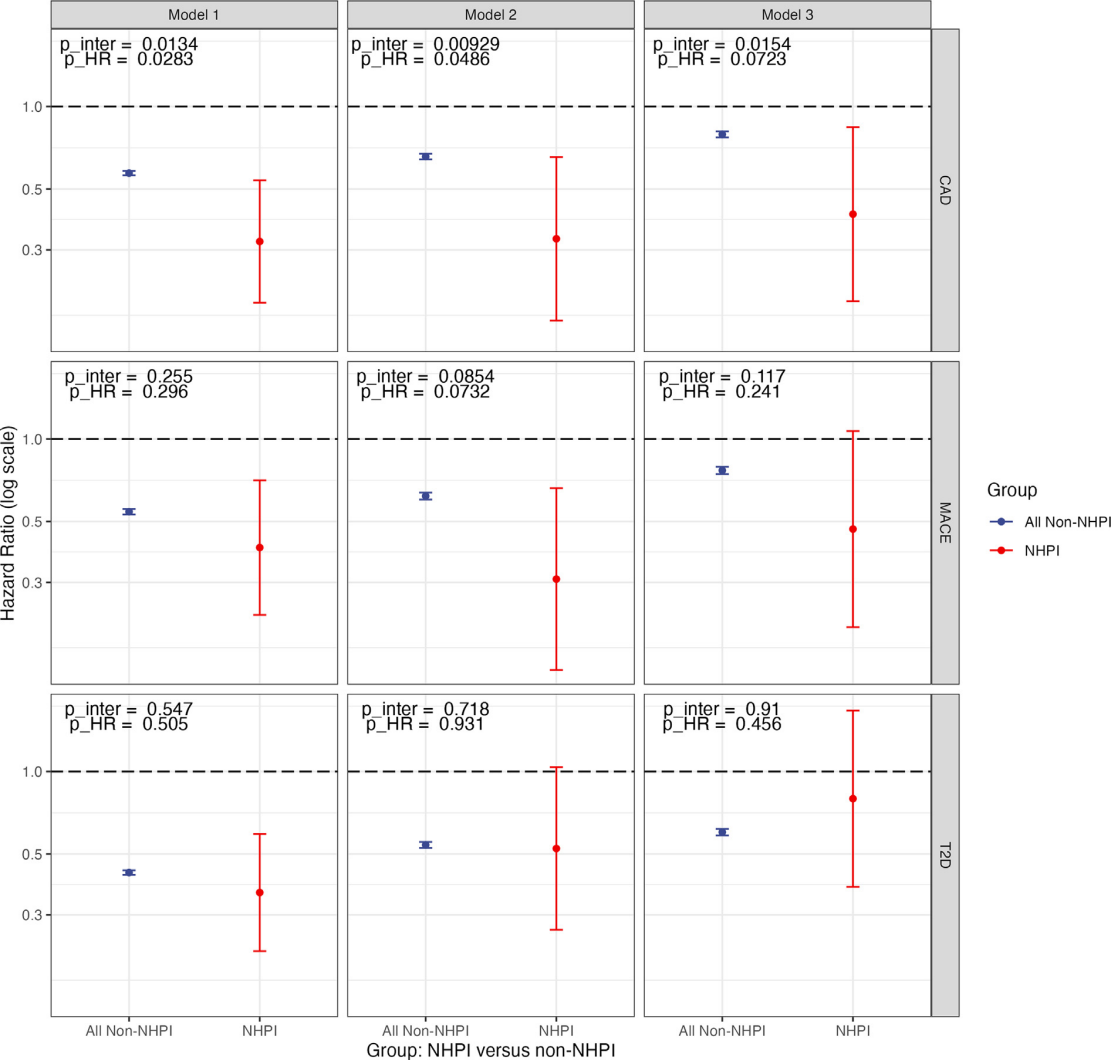
Summary Figure for Cox Proportional Hazards Models Compares HR for coronary artery disease (CAD, top), major adverse cardiovascular events (MACE, middle), and type 2 diabetes (T2D, bottom) between NHPI and all non-NHPI individuals in the National Institutes of Health *All of Us* Research Program database. Model 1, unadjusted univariate; Model 2, adjusted for LDL-C and triglycerides; Model 3, adjusted for LDL-C, triglycerides, sex, SBP, BMI, T2D diagnosis, statin prescription, antihypertensive drug prescription, and smoking history. Error bars, 95% CIs; y-axis is log scale. *P* values for interaction are reported directly from the model; HRs were compared using a Wald test based on the *z* statistic, and *P* values (p_HR) were reported. CIs for non-NHPI samples are vanishingly small and may not be visible to the reader. CAD = coronary artery disease; MACE = major adverse cardiovascular events; other abbreviations as in Figure 1.

We first considered the effect of HDL-C on the incidence of CAD across our 2 cohorts. Computing causal-specific proportional hazards curves under Model 1, we noted that the difference in median survival between high and low HDL-C was larger in the NHPI cohort than in the non-NHPI cohort, suggesting a potential difference in cardioprotective effect size (Figure 2). Model 1 confirmed this conclusion: there was a significant interaction (*P* = 0.0134) for NHPI status and HDL-C, and in direct comparison, the HR for high vs low HDL was 0.57 (95% CI: 0.56-0.58) in non-NHPI individuals, but a significantly lower 0.32 (95% CI: 0.19-0.54) among NHPIs (*P* = 0.0283, Wald test) (Figure 3, top row).

**Figure 4:**
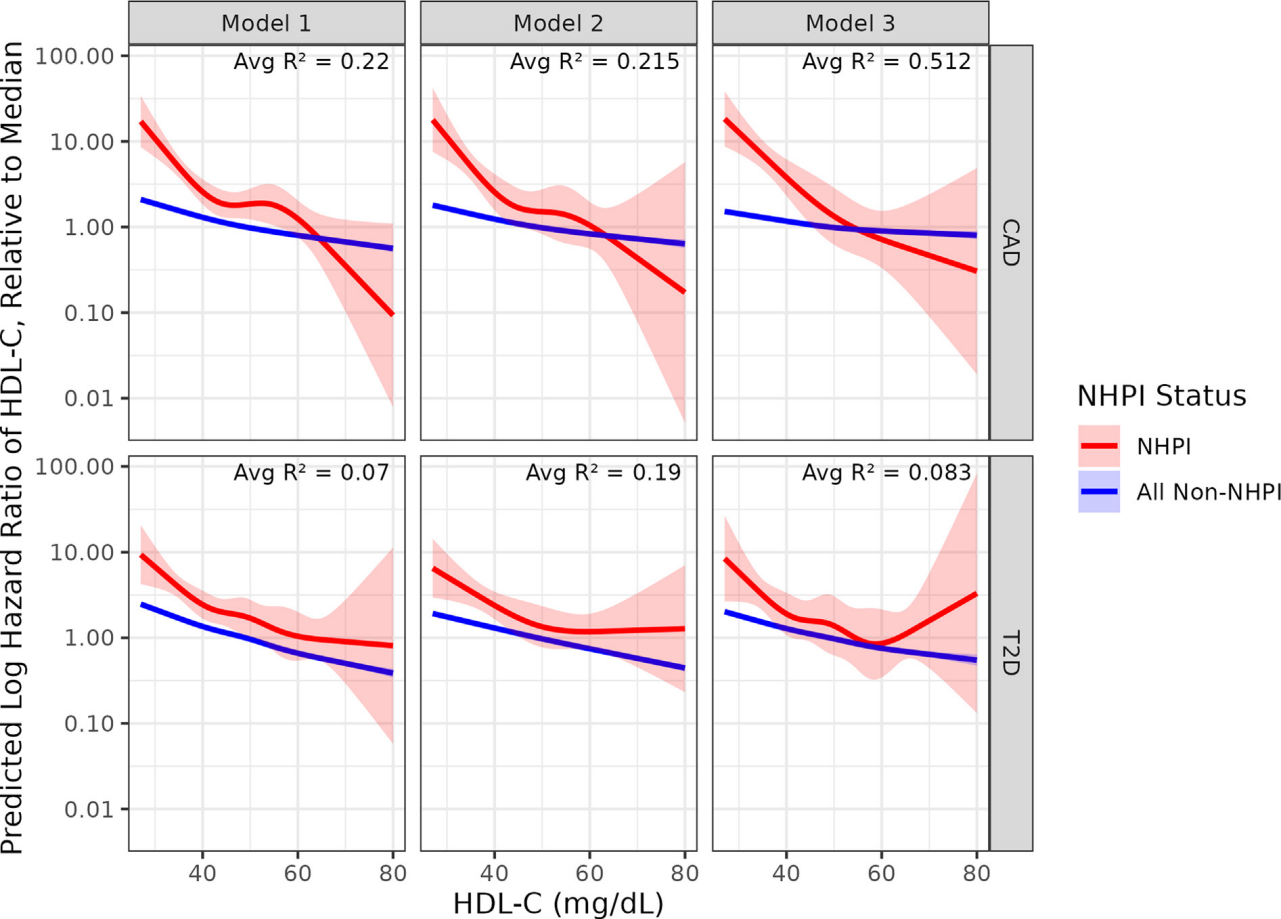
Restricted Cubic Splines Constructed for the Log HR of HDL-C at Each HDL-C Value, Relative to Median HDL-C for Models 1 to 3 for Coronary Artery Disease (CAD) and Type 2 Diabetes (T2D), Stratified by NHPI Status Abbreviations as in Figure 1.

When adjusting for the covariates in Models 2 and 3 (Figure 1), we found similar results. In Model 2, the interaction remained significant (*P* = 0.00929, Wald test), and the HR comparison was 0.66 (95% CI: 0.64-0.67) vs 0.33 (95% CI: 0.17-0.65) (*P* = 0.0486). In Model 3, the effect was weaker: 0.79 (95% CI: 0.77-0.81) vs 0.44 (95% CI: 0.19-0.84) (*P* = 0.0723, Wald test), but with a significant interaction (*P* = 0.0154) (Figure 3, top row).

We repeated the same analysis for MACE, across Models 1, 2, and 3. In Model 1, the HRs were 0.54 (95% CI: 0.53-0.56) in non-NHPI individuals, but a stronger 0.44 (95% CI: 0.23-0.71) in NHPI, though both the interaction (*P* = 0.255) and the HR difference (*P* = 0.2977, Wald test) were nonsignificant. We observed a weak interaction under Model 2 (*P* = 0.0854) with HRs 0.62 (95% CI: 0.60-0.64) vs 0.31 (95% CI: 0.14-0.66) (*P* = 0.0727, Wald test) and Model 3 was again nonsignificant: 0.77 (95% CI: 0.74-0.79) vs 0.47 (95% CI: 0.21-1.07) (*P* = 0.117 for interaction, *P* = 0.234 for HR comparison, Wald test) (Figure 3, center row).

We next extended our analysis to T2D, using the same Cox regression approach, and the same covariate sets, minus T2D diagnosis and usage of statins and antihypertensives in Model 3. Here, the results were null across the board. Under Model 1, the difference in HR was 0.43 (95% CI: 0.42-0.44) for non-NHPI vs 0.36 (95% CI: 0.22-0.59) for NHPI. Under Models 2 and 3, the HR was either similar or higher among NHPIs compared to non-NHPIs: 0.54 (95% CI: 0.53-0.55) vs 0.52 (95% CI: 0.26-1.04) and 0.60 (95% CI: 0.59-0.62) vs 0.8 (95% CI: 0.38-1.67). The interaction terms for NHPI and HDL-C on T2D were all nonsignificant (Figure 3, bottom row).

### Restricted cubic spline analysis

To better illustrate how continuously varying levels of HDL-C influence CAD and T2D risk among NHPI vs non-NHPI individuals, we applied a restricted cubic spline approach rather than using binary cutoffs for high or low HDL-C. The optimal number of knots and penalty terms were determined through an extensive validation process, testing 3 to 5 knots and penalties ranging from 0.1 to 10. This optimization aimed to maximize model discrimination while avoiding overfitting, as assessed through bootstrap-validated *D*_*xy*_ statistics (Supplemental Text 1).

The resulting spline models (Figure 4) revealed distinct patterns in the log HRs across HDL-C levels. For CAD, NHPI individuals exhibited a more pronounced response to HDL-C variation compared to non-NHPI individuals. At low HDL-C levels (40 mg/dL), NHPI individuals showed approximately a 10-fold higher log HR relative to the median, while non-NHPI individuals showed roughly a 5-fold increase. This relationship became more nuanced at higher HDL-C levels, with the NHPI population showing a steeper decline in risk, particularly above 60 mg/dL, where their log HR dropped below that of the non-NHPI population (Figure 4).

For T2D, the relationship was more complex, particularly in Model 3, which showed a U-shaped response for NHPI individuals. The log HR was highest at low HDL-C levels, reached its minimum around 60 to 65 mg/dL, and showed a slight uptick at higher levels. This nonlinear pattern suggests an optimal HDL-C range for T2D risk reduction in the NHPI population, beyond which additional increases in HDL-C may not confer further benefits (Figure 4).

The models’ explanatory power, as indicated by the R^2^ values for the CAD models (0.22, 0.215, and 0.312) and for the T2D models (0.07, 0.12, and 0.083), demonstrates that HDL-C levels account for a meaningful portion of risk variation, particularly for cardiovascular outcomes. The higher R^2^ values in the CAD models suggest that HDL-C may be a more robust predictor of cardiovascular risk than diabetes risk.

## Discussion

This study reports the uniquely strong association of HDL-C levels with reduced risk of CAD, and attenuated-to-null effects for MACE and T2D among NHPIs. Our Cox proportional hazards models demonstrate that high levels of HDL-C are associated with a reduced risk of CAD and MACE but not T2D in NHPIs compared to non-NHPIs. These results are robust across adjustment for 3 sets of covariates, including LDL-C, triglycerides, sex, BMI, SBP, statin and antihypertensive drug prescriptions, smoking history, and alcohol usage. Furthermore, through our spline models, we demonstrate novel nonlinear associations of HDL-C levels with these diseases in NHPI populations, contributing to a growing body of work on the nonlinear effects of HDL, including in individuals of non-European ancestry.^28^, ^29^, ^30^, ^31^, ^32^, ^33^, ^34^

### Study Limitations

Our approach does have several key limitations, mostly stemming from the limitations of the *All of Us* data set. All-cause mortality is a competing risk for each of our outcomes, making a competing risk, rather than causal-specific hazards, model theoretically more appropriate; *All of Us*, however, does not extract participant deaths from the EHR, and anonymization for privacy purposes inexorably precludes any cross-referencing with other databases. Therefore, individual HRs are likely somewhat overestimated, but this overestimation should be consistent across both the NHPI and non-NHPI cohorts.

Our analysis in this study was also limited by the relatively small sample of NHPI individuals in *All of Us*, though it is among the largest currently available to researchers.^17^ As a result, the proportional hazards regressions retained a substantial degree of uncertainty, particularly with the constraint of additional model parameters, and the range of HDL-C values over which we were able to fit spline curves was narrowed to modestly high or low HDL-C (about 20-80 mg/dL). Our spline models therefore do not provide a good picture of the effects of extremely high or extremely low HDL-C levels on CVD/T2D risk in NHPI, ranges in which prior work has documented strong increases in CVD/T2D risk.^6^^,^^28^^,^^30^^,^^32^^,^^34^ While this narrowing was necessary in order to ensure meaningful cross-group comparisons, it does exclude approximately 20% of observations in both populations.

These limitations of sample size and data quality underscore the importance of expanding the inclusion of NHPI in available research biobanks; such efforts should enable the discovery of many more ancestry- and environment-specific effects like the ones described in this study and should ultimately broaden the promise of precision medicine.

### Future directions

This study also did not attempt to address potential underlying mechanisms for our biostatistical observations about HDL-C, but we propose several possibilities. Most likely, NHPI populations carry unique genetic factors associated with historic oceanic lifestyles. Lipid genome-wide association studies have uncovered ancestry-specific variants associated with lipid levels and metabolism,^35^, ^36^, ^37^, ^38^ which when combined with ongoing research into the functional biology and molecular diversity of HDL-C particles themselves,^1^ suggests that NHPI populations may have unique genetic variants that might, for example, strengthen the anti-inflammatory and antiatherogenic power of HDL-C. Indeed, a published polygenic score (PGS) for HDL-C levels from the Global Lipids Genetics Consortium performed exceptionally poorly among the NHPI individuals genotyped in *All of Us*, suggesting an HDL genetic architecture different from the populations the PGS was originally trained on (n = 660) (Supplemental Figure 6).^38^

Despite its limitations, we believe our study points the way toward further research on the performance of traditional risk prediction models and biomarkers among NHPI populations. Given how few NHPI patients tend to be included in the development of these models, it is likely that they will perform more poorly when applied to NHPI populations. Future work should assess this hypothesis for a range of canonical biomarkers, including lipid levels.

This study has uncovered further research questions regarding why HDL-C confers the additional protective effect we observed, and we propose several related hypotheses. First, as our PGS analysis (see Supplemental Figure 6) indicates, the genetic architecture of HDL-C levels may be substantially different in NHPI, suggesting the existence of a genetic effect. Such a putative genetic effect might also be related to the much-debated (though recently doubted) thrifty gene hypothesis, which argues that famine cycles and open-ocean journeys may have positively selected variants associated with fat conservation.^39^

Second, unique environmental factors may account for this effect. Decades of epidemiological research have identified diet and lifestyle as causes of the severe epidemic of obesity, diabetes, and CVD among NHPI populations, and so some interaction of these factors with lipid metabolism may also account for the observed effect.^40^, ^41^, ^42^, ^43^, ^44^ A gene-environment interaction, for example, a genetic effect activated only in the presence of an NHPI-specific environment, may also exist. Future work should seek to address these questions. Taken together, these approaches will yield a broader, more inclusive understanding of the relationships between genetics, environment, lipid levels, and cardiometabolic disease, and will form the basis of risk models and clinical guidelines that account for differences between NHPIs and other populations.

Taken together, these approaches will yield a broader, more nuanced understanding of the relationships between genetics, environment, lipid levels, and cardiometabolic disease, and will form the basis of risk models and clinical guidelines that account for differences between NHPIs and other populations. Clinically, such observations underscore the need to view traditional risk equations, currently derived mainly from European ancestry populations, with caution when caring for NHPI individuals, whose risk may be underestimated or overestimated if standard cutoffs are applied.^28^ Even so, the central importance of managing established factors like LDL, blood pressure, and glycemic control remains unchanged; for NHPI patients, these measures can be complemented by interventions that favorably shape HDL-C, such as enhanced physical activity and smoking cessation.^43^

Simultaneously, it remains critical to address the disproportionately high burden of cardiometabolic disease in NHPI communities through more precise research and ancestry-specific analyses.^16^ By evaluating biomarkers like HDL-C within these groups, clinicians and investigators can better refine risk stratification tools and tailor preventive strategies.^41^ Ultimately, these efforts promote greater improvement in cardiovascular and metabolic health outcomes, ensuring that individuals from all backgrounds benefit from the progress made in precision medicine.^44^

## Conclusions

This study reveals for the first time the unique protective dynamics of elevated HDL-C levels against CAD, with weaker effects for MACE, and T2D among NHPI populations, with implications for clinical assessment. With such insights in mind, more individually tailored steps toward eliminating the substantial cardiovascular health differences faced by NHPI communities may be possible.

Perspectives **COMPETENCY IN MEDICAL KNOWLEDGE 1:** Our investigation demonstrates that HDL-C’s protective association against CAD is significantly stronger in NHPI populations than in the general population, with more modest differential effects for MACE and T2D.

**COMPETENCY IN MEDICAL KNOWLEDGE 2:** While standard preventive strategies remain essential, clinicians caring for NHPI patients should consider placing additional emphasis on interventions that favorably modify HDL-C levels, such as physical activity, dietary modifications, and smoking cessation, potentially at different thresholds than those applied to the general population.

**COMPETENCY IN MEDICAL KNOWLEDGE 3:** These findings underscore the importance of targeted research and interventions for NHPI communities, who face disproportionate cardiometabolic disease burdens. Biomarker studies across diverse populations are essential for developing precision medicine approaches and reducing health disparities.

**TRANSLATIONAL OUTLOOK 1:** These findings challenge the practice of applying uniform risk assessment tools across all populations. Clinical risk algorithms and treatment thresholds developed primarily from European ancestry cohorts may inadequately capture cardiovascular risk in NHPI individuals, potentially leading to missed opportunities for early intervention.

**TRANSLATIONAL OUTLOOK 2:** The unique nonlinear risk dynamics of HDL-C in NHPIs suggests that population-specific reference ranges may be more appropriate than universal cutoffs. For NHPIs, maintaining HDL-C levels above 60 mg/dL appears particularly beneficial for CAD risk reduction.

**TRANSLATIONAL OUTLOOK 3:** Future research should investigate the biological mechanisms underlying this enhanced protective effect, including potential genetic adaptations related to historical oceanic lifestyles, unique HDL particle composition, or gene-environment interactions specific to NHPI populations.

## Funding support and author disclosures

Research reported in this publication was supported by the 10.13039/100000062National Institute of Diabetes and Digestive and Kidney Diseases (NIDDK) of the National Institutes of Health (Bethesda, Maryland) (R01DK132090 to Dr Khomtchouk). The authors have reported that they have no relationships relevant to the contents of this paper to disclose.
