# Supplementary material for: HDL Cholesterol Is Remarkably Cardioprotective Against Coronary Artery Disease in Native Hawaiians and Pacific Islanders
Source: JACC Adv. 2025 May 2;4(6):101741. doi: 10.1016/j.jacadv.2025.101741 (PMC12124629; doi:10.1016/j.jacadv.2025.101741)
Supplement: Supplementary data [file mmc1.pdf]

Szatrowski, Maggio, & Khomtchouk (2025)
SUPPLEMENTARY MATERIALS

Table S1
Figure S1
Figure S2
Figure S3
Figure S4
Supplementary Text ST1
Figure S5
Figure S6
Figure S7
Figure S8

Disease	ICD/SNOMED Codes
Coronary artery disease (CAD)	ICD9: 410, 411, 412, 413, 414<br>ICD10: I20, I22, I24, I25
Myocardial infarction (MI)	ICD9: 410, 412<br>ICD10: I21, I22, I25.2
Major adverse cardiovascular events (MACE)	ICD9: 410, 412 (MI); 433, 434 (ischemic stroke)<br>ICD10: I21, I25.2 (MI); I63, I65, I66 (ischemic stroke); SNOMED 95281009 (sudden cardiac death)
Type 2 diabetes (T2D)	ICD10: E11, IC9: 250.00, .00, .10, .20, .30, .40, .50, .60, .70, .80, .90, .02, .12, .22, .32, .42, .52, .62, .72, .82, .92, SNOMED 44054006

Table S1. Disease definitions by ICD10, ICD9, and SNOMED codes, as applied in the NIH *All of Us* Research Program biobank.

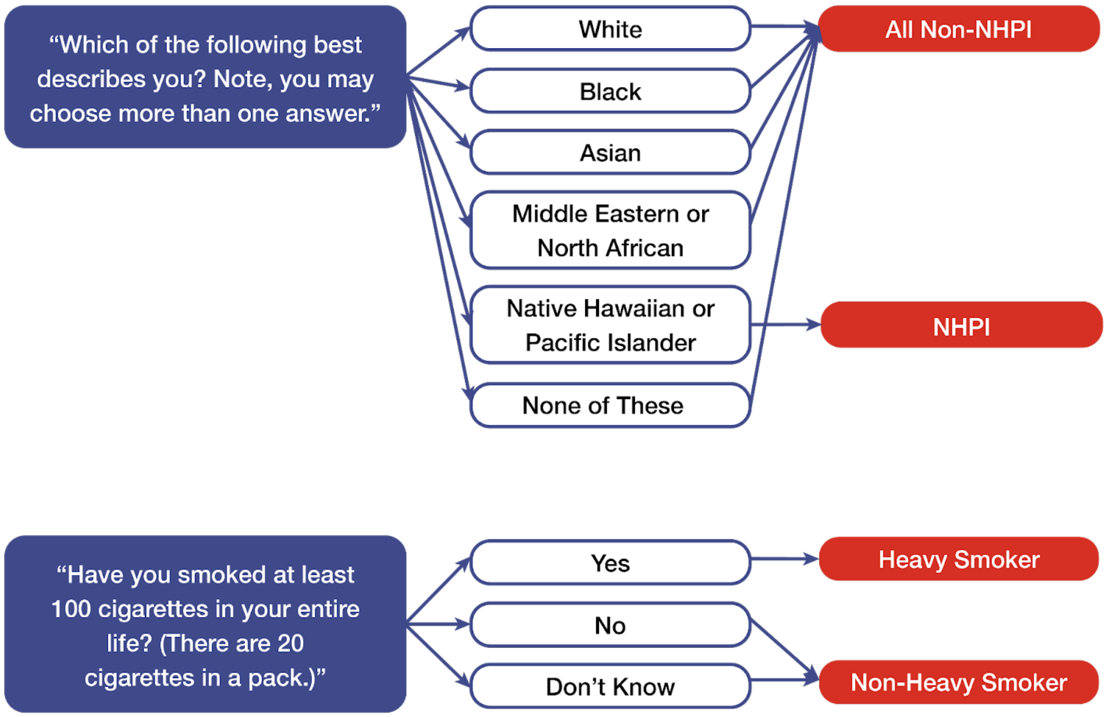

Figure S1. NIH *All of Us* Research Program survey questions used to stratify study participants.

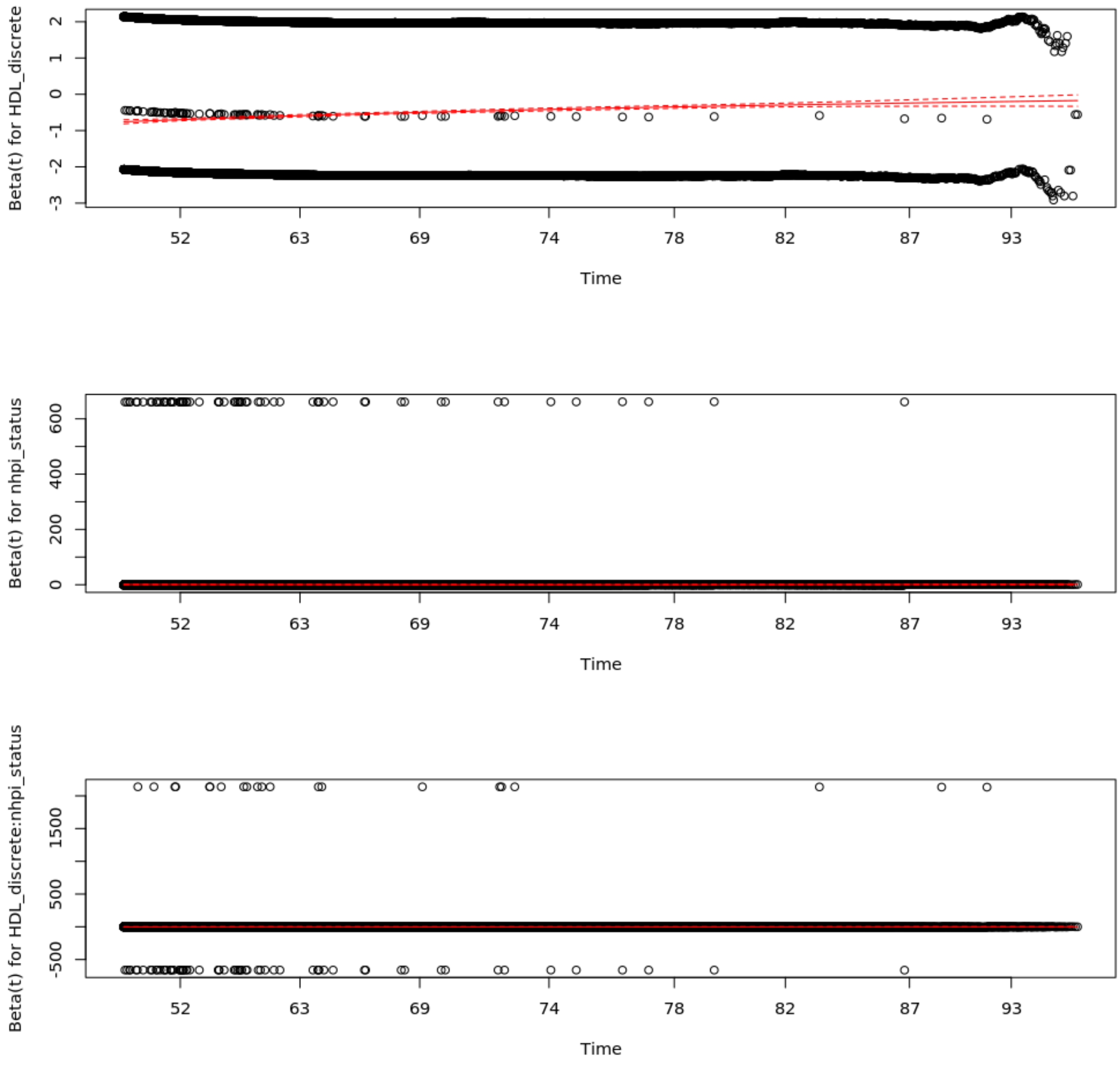

Figure S2. Test of proportional hazards assumptions for CAD Model 1.

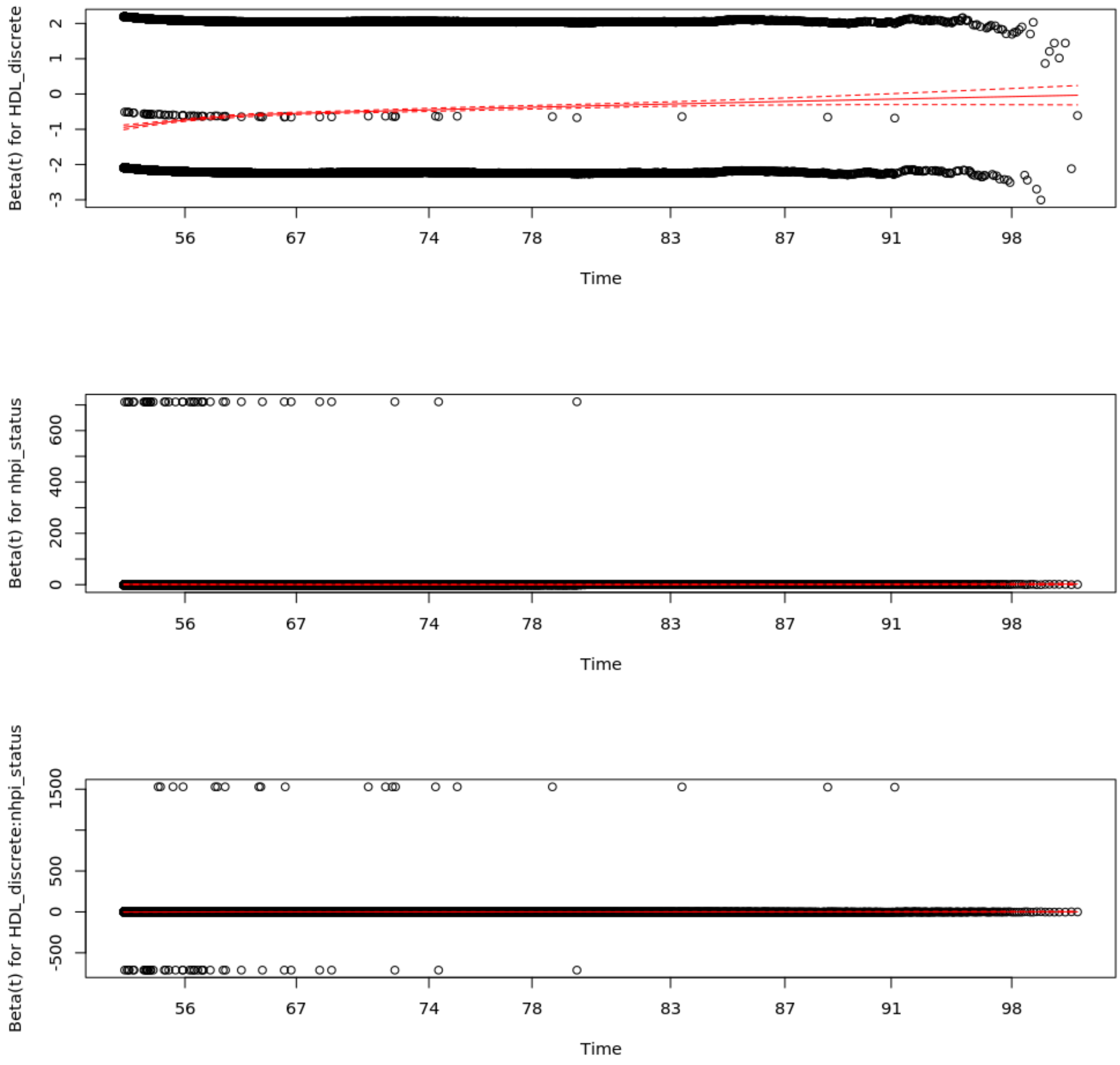

Figure S3. Test of proportional hazards assumptions for MACE Model 1.

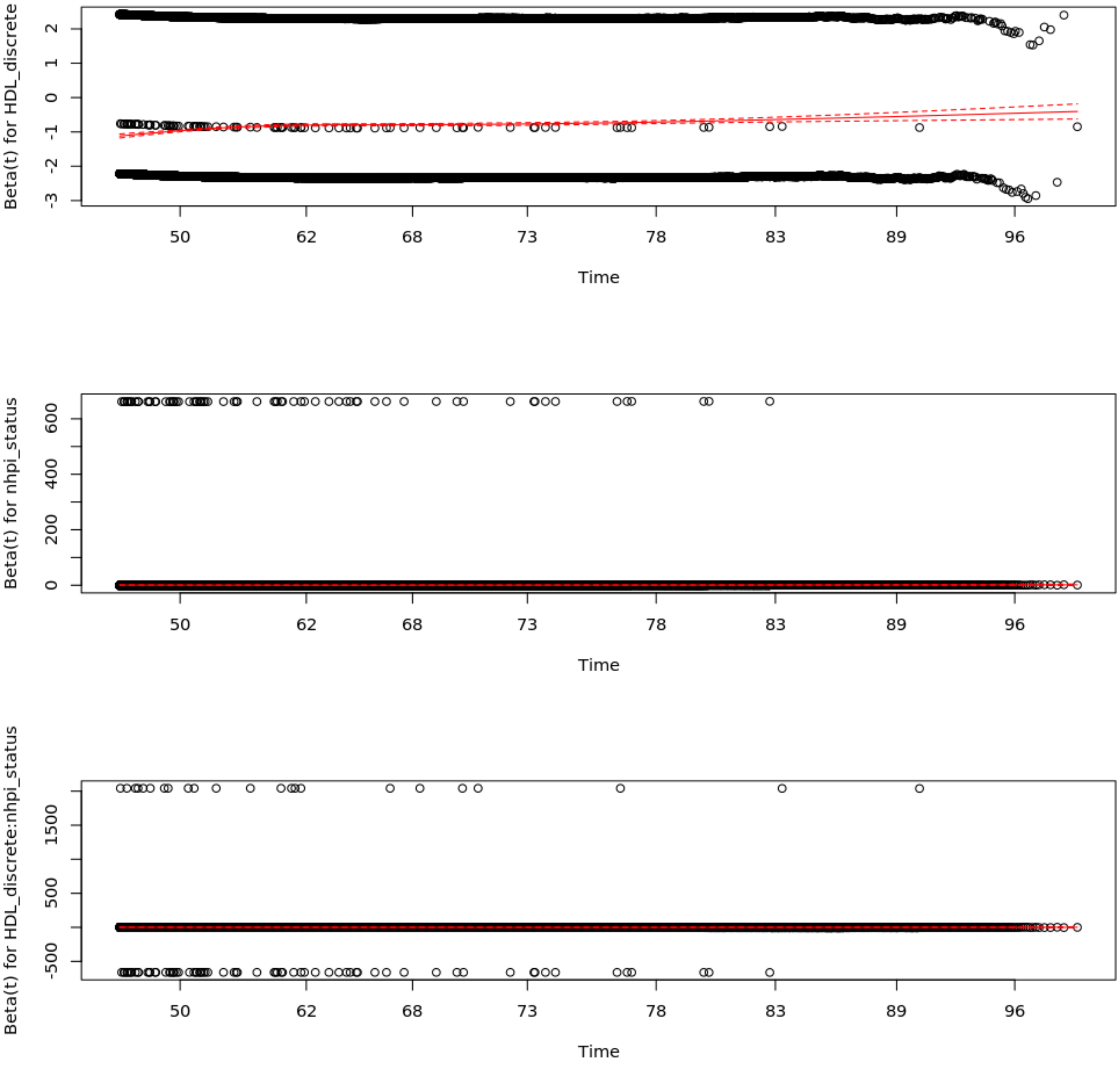

Figure S4. Test of proportional hazards assumptions for T2D Model 1.

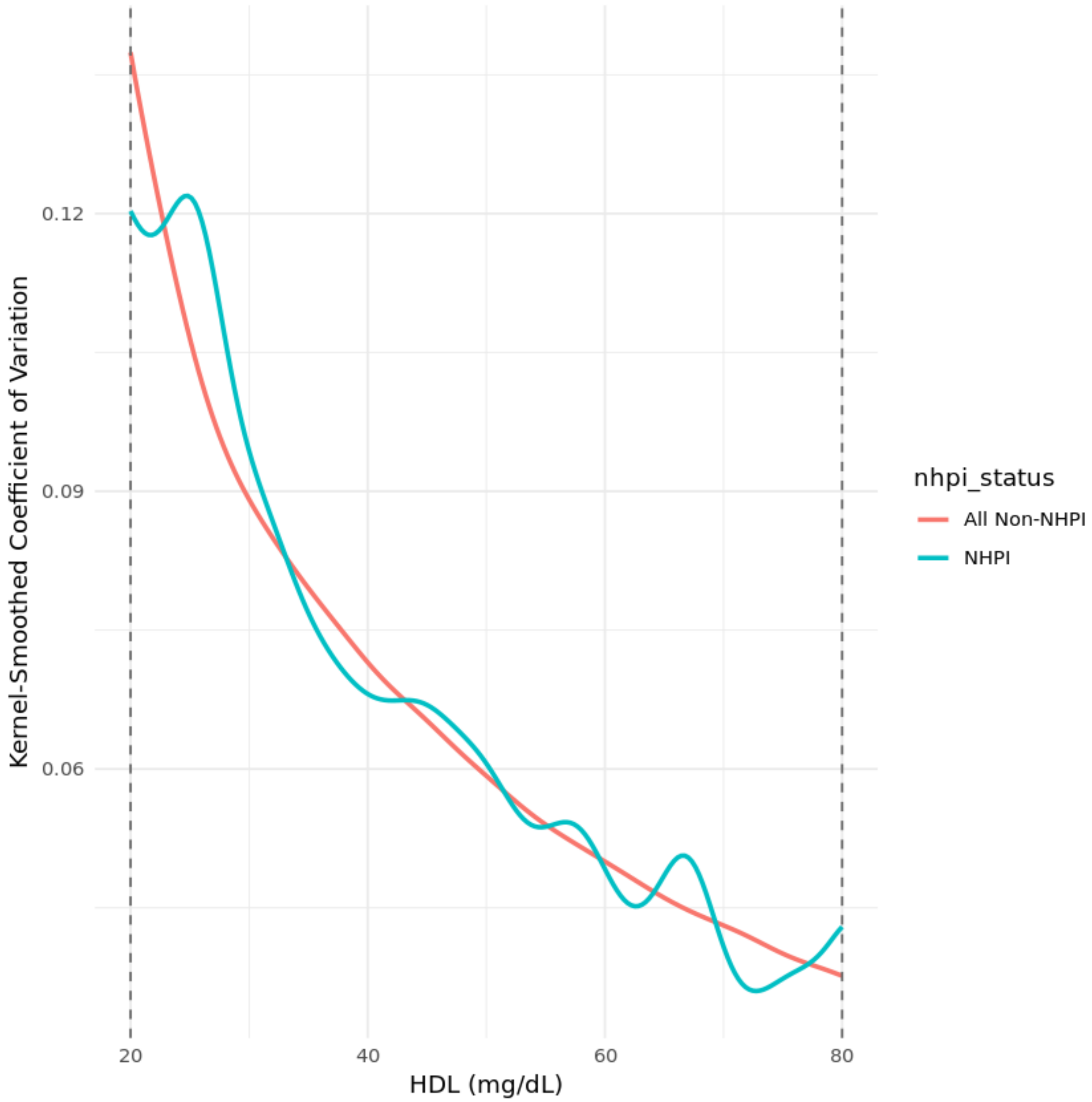

Figure S5. Kernel-smoothed coefficient of variation (CV) for HDL-C (mg/dL) across the 20–80 mg/dL range, stratified by NHPI status.

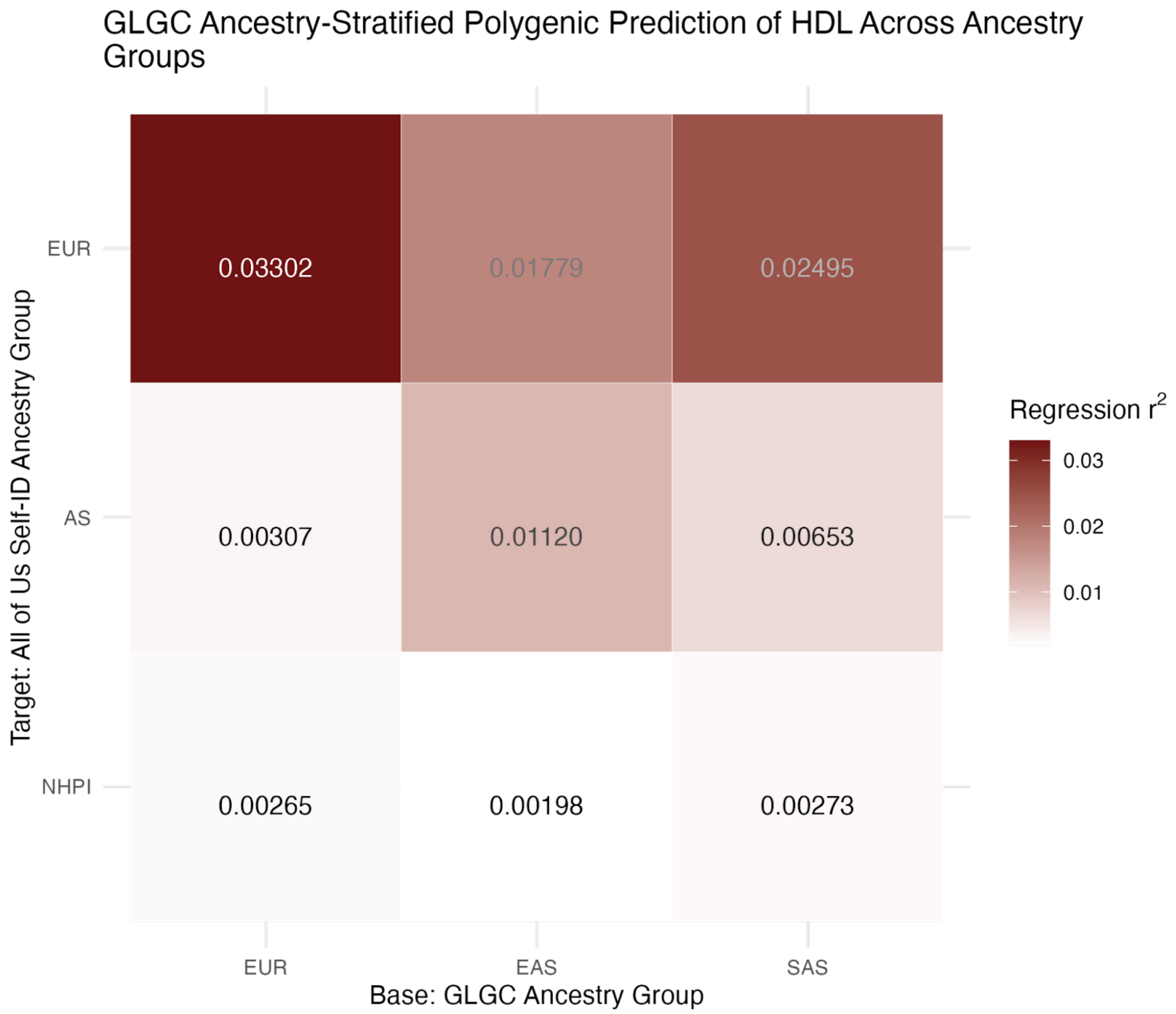

Figure S6. Performance (as r^2) of selected ancestry-specific polygenic risk scores for HDL-C levels.

Supplementary Text ST1: Spline Tuning and Model Selection.

To identify optimal spline parameters for our restricted cubic spline (RCS) analysis of CAD and T2D, we performed a systematic grid search of the number of knots (3, 4, or 5) and spline penalty (ranging from 0.1 to 10). We fit each candidate RCS with a Cox proportional hazards framework and evaluated model discrimination using Harrel's D_{xy}. The scales in figures **S7** and **S8** indicate higher D_{xy} values, signifying stronger concordance between predicted and observed outcomes. We selected the knots and penalty configuration yielding the highest D_{xy} values for the final spline models used in **Figure 4.**

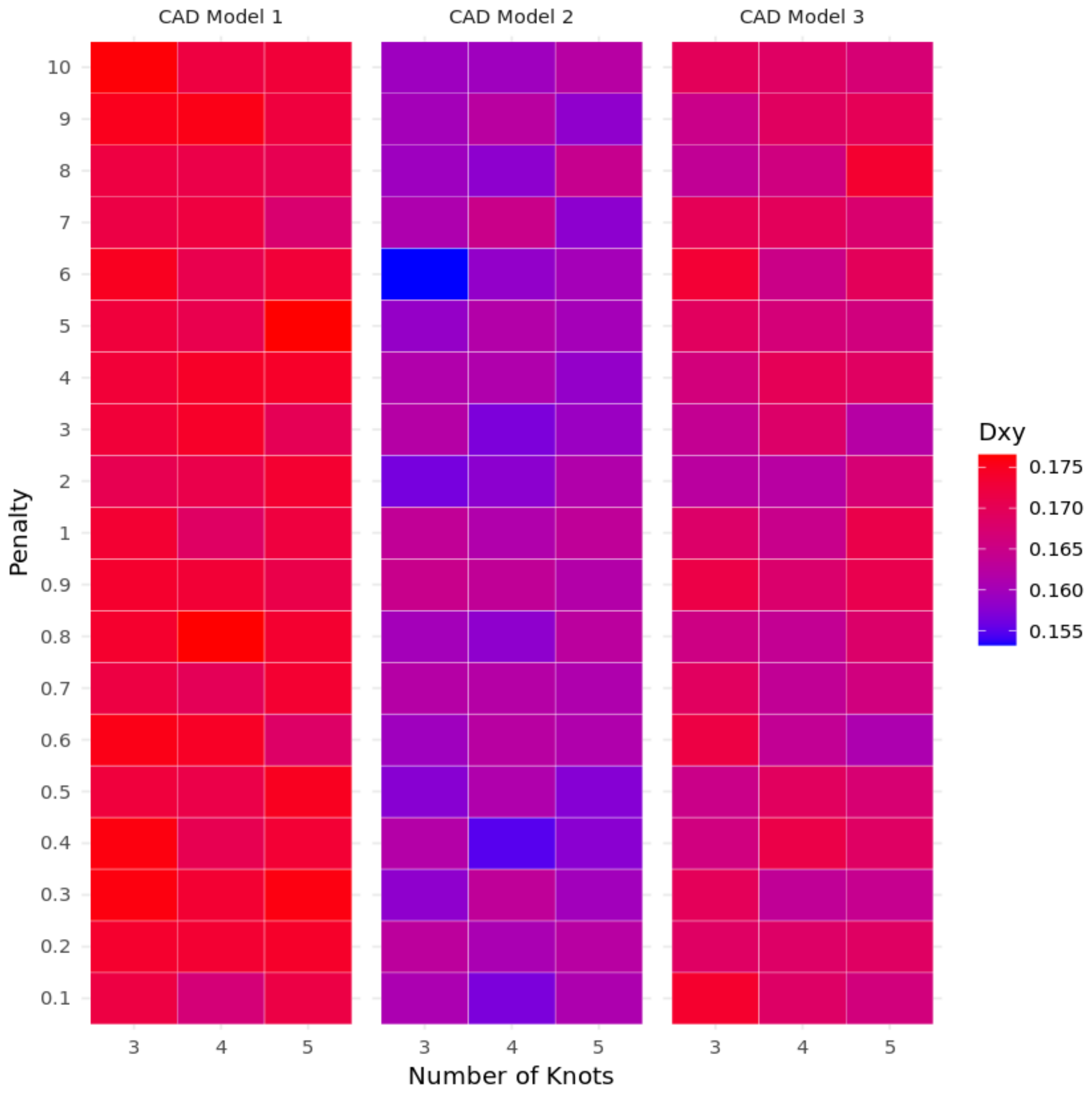

Figure S7. CAD Tuning Heatmap: Knots vs. Penalty, Stratified by Model.

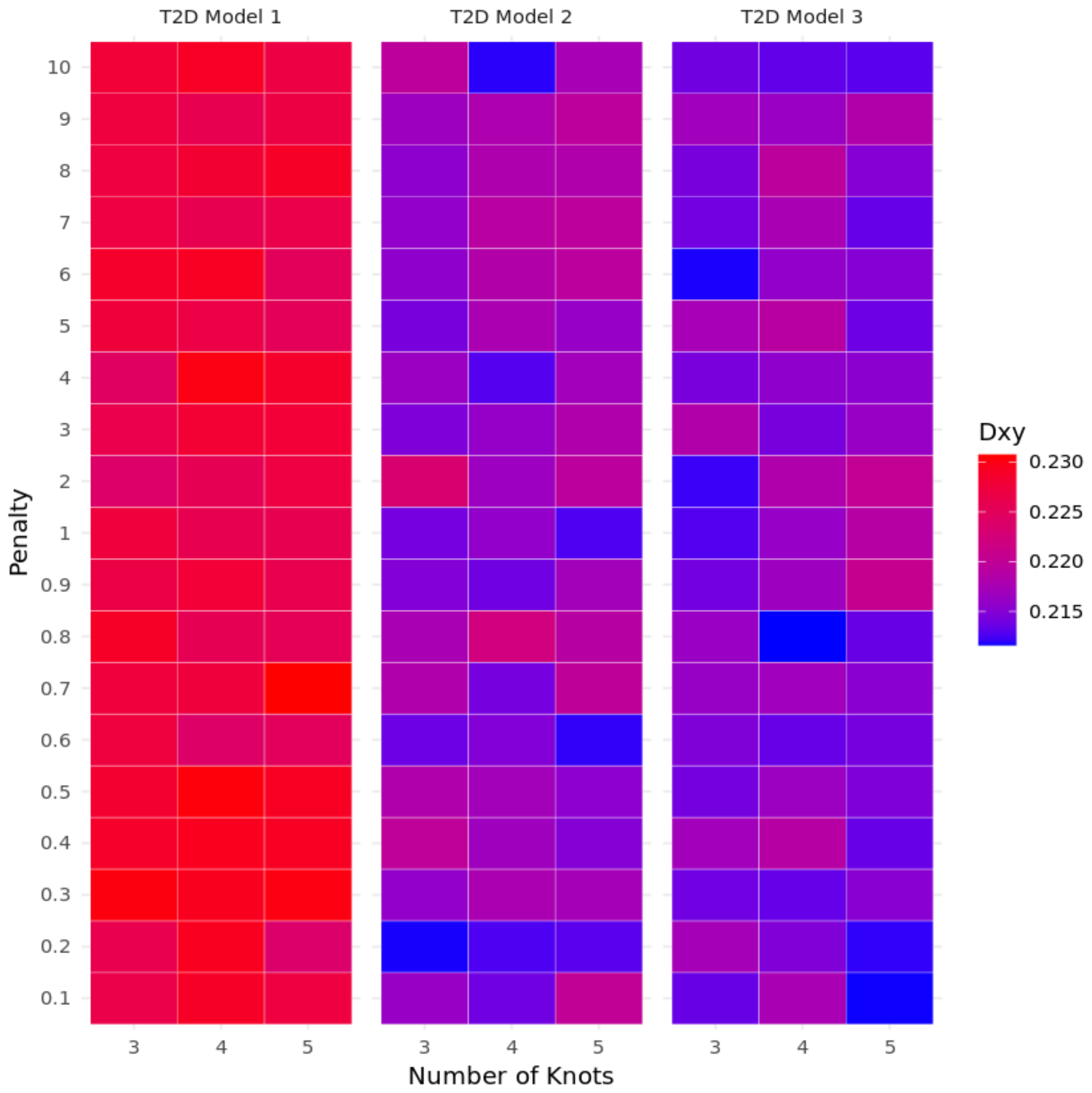

Figure S8. T2D Tuning Heatmap: Knots vs. Penalty, Stratified by Model.
